# Identification of a Novel Stem Cell Subtype for Clear Cell Renal Cell Carcinoma Based on Stem Cell Gene Profiling

**DOI:** 10.3389/fonc.2021.758989

**Published:** 2021-11-29

**Authors:** Hongzhi Wang, Hanjiang Xu, Quan Cheng, Chaozhao Liang

**Affiliations:** ^1^ Department of Urology, The First Affiliated Hospital of Anhui Medical University, Hefei, China; ^2^ Institute of Urology, Anhui Medical University, Hefei, China; ^3^ Anhui Province Key Laboratory of Genitourinary Diseases, Anhui Medical University, Hefei, China; ^4^ Department of General Surgery, the First Affiliated Hospital of Nanjing Medical University, Nanjing, China

**Keywords:** clear cell renal cell cancer (ccRCC), stem cell, subtype, gene expression, immune microenvironment

## Abstract

Clear cell renal cell carcinoma (ccRCC) is the most common subtype of renal cancer and is characterized by high rates of metastasis. Cancer stem cell is a vital cause of renal cancer metastasis and recurrence. However, little is known regarding the change and the roles of stem cells during the development of renal cancer. To clarify this problem, we developed a novel stem cell clustering strategy. Based on The Cancer Genome Atlas (TCGA) and the International Cancer Genome Consortium (ICGC) genomic datasets, we used 19 stem cell gene sets to classify each dataset. A machine learning method was used to perform the classification. We classified ccRCC into three subtypes—stem cell activated (SC-A), stem cell dormant (SC-D), and stem cell excluded (SC-E)—based on the expressions of stem cell-related genes. Compared with the other subtypes, C2(SC-A) had the highest degree of cancer stem cell concentration, the highest level of immune cell infiltration, a distinct mutation landscape, and the worst prognosis. Moreover, drug sensitivity analysis revealed that subgroup C2(SC-A) had the highest sensitivity to immunotherapy CTLA-4 blockade and the vascular endothelial growth factor receptor (VEGFR) inhibitor sunitinib. The identification of ccRCC subtypes based on cancer stem cell gene sets demonstrated the heterogeneity of ccRCC and provided a new strategy for its treatment.

## Introduction

Renal cancer accounts for 3% of all adult malignancies worldwide, and its incidence have been increasing in recent years. Clear cell renal cell carcinoma (ccRCC) is the most common subtype of renal cancer, jeopardizing 70%–80% of renal cell carcinoma (RCC) patients. Over 30% of ccRCC patients have distant metastasis at the time of diagnosis, and one-third of patients with localized ccRCC will develop metastasis after nephrectomy (1–3). The 5-year survival rate of localized ccRCC is about 65%; however, it drops to 10%–20% after cancer metastasis (4). Surgery remains the major approach for the treatment of localized ccRCC; nevertheless, novel therapeutic strategies are urgently needed for metastatic patients.

Significant achievements have been made in treating advanced ccRCC in the last two decades, such as the application of tyrosine kinase and mammalian target of rapamycin (mTOR) inhibitors or monoclonal antibodies against vascular endothelial growth factor (VEGF) (5, 6). Combination therapy with these inhibitors prolonged the life span of patients. However, most tumors will progress within 2 years. Recently, new approaches for boosting the immune response to renal tumors with immune checkpoint inhibitors, which block programmed cell death protein-1 (PD-1) or cytotoxic T-lymphocyte-associated protein 4 (CTLA-4) on T cells, have shown promising effects in a subset of patients (7). Fundamentally, improving the outcome of renal cancer patients will require personalized treatment strategies specific to the biological characteristic of each tumor.

Stem cells are defined as cells with the ability to self-renew and differentiate into mature cells of a particular tissue (8). Cancer stem cells (CSCs) are a subpopulation of cancer cells with a higher self-renewal ability and the ability to reproduce the heterogeneity of tumors (9). CSCs have been characterized in various cancers and have been proven to contribute to drug resistance, tumor recurrence, and distant metastasis; however, the situation in kidney cancer remains obscure (10–12). Some studies have indicated that targeting the Notch, Hedgehog, and Wnt signaling pathways could inhibit the self-renewal and pluripotency ability of ccRCC cancer stem cells (13, 14). DKK3 and Notch3, which are members of the Wnt and Notch pathways, have been proven to be indicators of the prognosis of renal cancer patients (13). IL8/CXCR1 signaling was proven to promote the sphere formation and self-renewal capability of renal tumor cells (15). Both IL8 and CXCR1 are significantly correlated with patient survival. These studies indicated that the genes expressed in renal CSCs (RCSCs) could be effective prognostic factors. However, the functional significance and the prognostic value of stem cell-related genes in ccRCC are still scarcely investigated and need to be further clarified.

In the present study, we aimed to identify the subclasses of ccRCC with different CSC properties based on the expressions of stem cell-related genes. We divided ccRCC into three clusters with different features and prognosis. In addition, we proved the stability and reliability of this clustering with an independent dataset using the unsupervised clustering method. Moreover, we comprehensively analyzed the prognosis of the RCSC subtypes, relationship with immune cells and genes, the sensitivity of the immune checkpoint inhibitor treatment, and potential changes in the biological process. Classification of the stem cell gene-related subtypes may contribute to formulating the optimal treatment for renal cancer patients.

## Results

### NMF Identifies Three Subclasses in ccRCC

**Figure 1:**
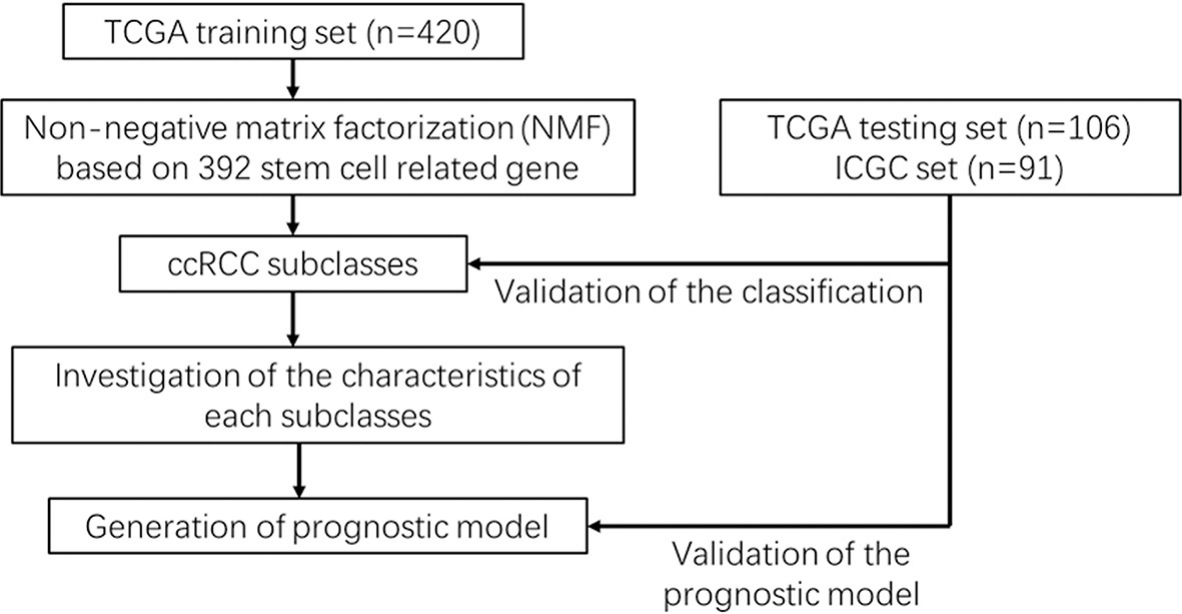
Workflow chart.

**Figure 2:**
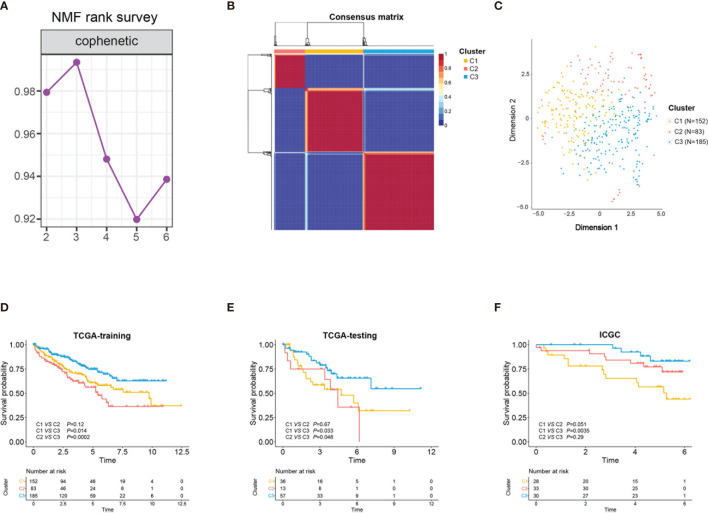
Identification of three stem cell subtypes using non-negative matrix factorization (NMF) consensus clustering in clear cell renal cell carcinoma (ccRCC) The Cancer Genome Atlas (TCGA) training cohort. **(A)** NMF clustering using 392 stem cell-associated genes. Cophenetic correlation coefficients for *k* = 2–6 are shown. **(B)** Consensus heatmap for ccRCC samples when *k* = 3. **(C)** *t*-Distributed stochastic neighbor embedding (t-SNE) analysis supported the stratification into three stem cell subtypes. *Dots with different colors* represent different samples in the subclasses. **(D–F)** Overall survival of the three subclasses (C1, C2, C3) in TCGA training and testing sets and the International Cancer Genome Consortium (ICGC) cohort.

An analysis flowchart was designed to systematically depict our study (**Figure 1**). Three hundred and ninety-two stem cell-related genes were enrolled for subsequent non-negative matrix factorization (NMF) analysis. The training set comprising 263 ccRCC samples from The Cancer Genome Atlas (TCGA) was clustered based on the expressions of the aforementioned 392 candidate genes using NMF consensus clustering. Cophenetic correlation coefficients, dispersion, and silhouette were calculated to identify the best *k* value; *k* = 3 was proven to be the optimal number of clusters (three subclasses were assigned: C1, C2, and C3) (**Figure 2A**). Based on the present classification, the consensus heatmap showed sharp and crisp boundaries, indicating the applicability and robust clustering of these samples (**Figure 2B**). To validate the subtype classification, we conducted *t*-distributed stochastic neighbor embedding (t-SNE) to reduce the dimension of the features and found that samples in the same subclass generally gather in the same region. This indicates that the subclasses were mostly accordant with the t-SNE distribution patterns (**Figure 2C**). Additionally, to validate this classification, we performed an independent analysis on TCGA testing set and the International Cancer Genome Consortium (ICGC) dataset, the results of which also demonstrated that there were three distinct molecular subclasses. Based on the classification, a significant prognostic difference was observed in both TCGA testing set and the ICGC dataset (**Figures 2D–F**).

### Correlation of the ccRCC Subclass With Stem Cell-Related Signatures

**Figure 3:**
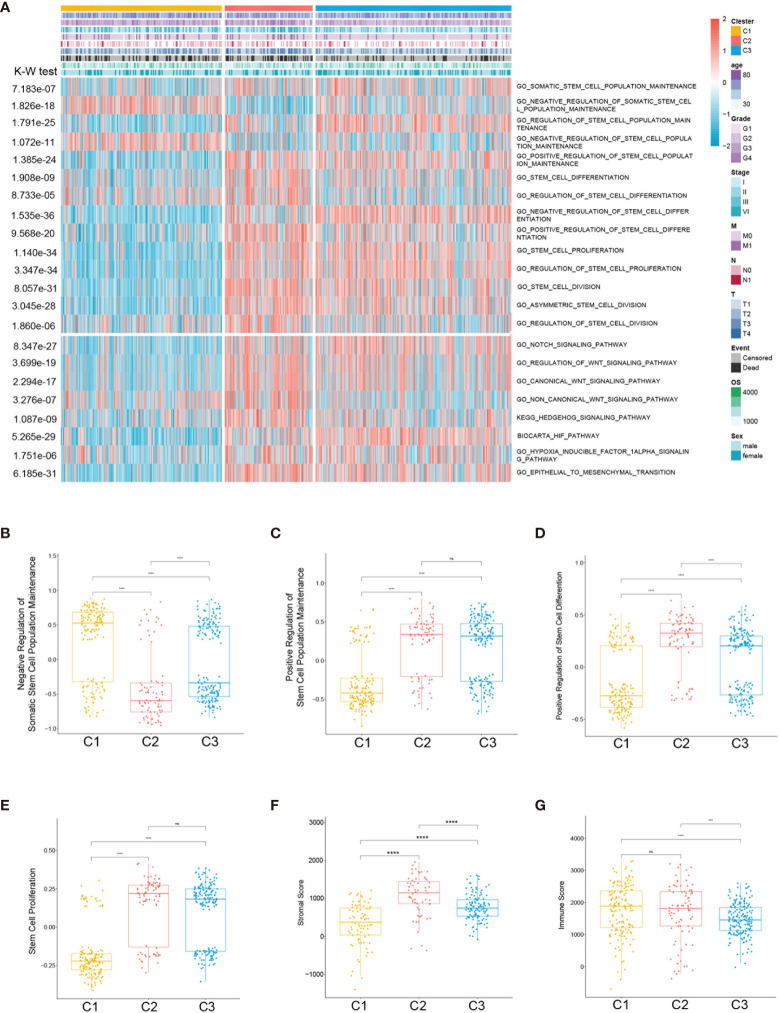
Association between the stem cell-associated signatures and the clear cell renal cell carcinoma (ccRCC) subclasses. **(A)** Heatmap of the specific stem cell-associated signatures. **(B–E)** Box plot of the signature scores for the stem cell-associated signatures distinguished by different subclasses. Box plot of the stromal **(F)** and immune **(G)** scores from estimates of the three subclasses. The *p*-values are labeled *above each box plot* with *asterisks*. *ns*, no significance. ***p < 0.001, ****p < 0.0001.

Considering that the clustering was based on stem cell-related genes, we further investigated whether the different subclasses had distinct stem cell characteristics. Firstly, 19 stem cell-related biological process scores were calculated using the GSVA R package based on TCGA training cohort. Three subtypes showed significantly different stem cell-related signatures and clinicopathological characteristics (**Figure 3A**). Similar trends were identified in the ICGC cohort (**Supplementary Figure S1**). The results showed that C1 was related to the negative regulation of stem cell maintenance (**Figure 3B**), while C2 and C3 were correlated with positive stem cell maintenance (**Figure 3C**). Moreover, the scores of the stem cell proliferation signatures were highest in C2 (**Figure 3E**). Compared with C3, the C2 subtype was characterized by higher stem cell proliferation and differentiation scores (**Figures 3D, E** **)**. Hence, we defined C1 as the stem cell excluded (SC_E) subclass, C2 as the stem cell activated (SC_A) subclass, and C3 as the stem cell dormant (SC_D) subclass.

**Figure 4:**
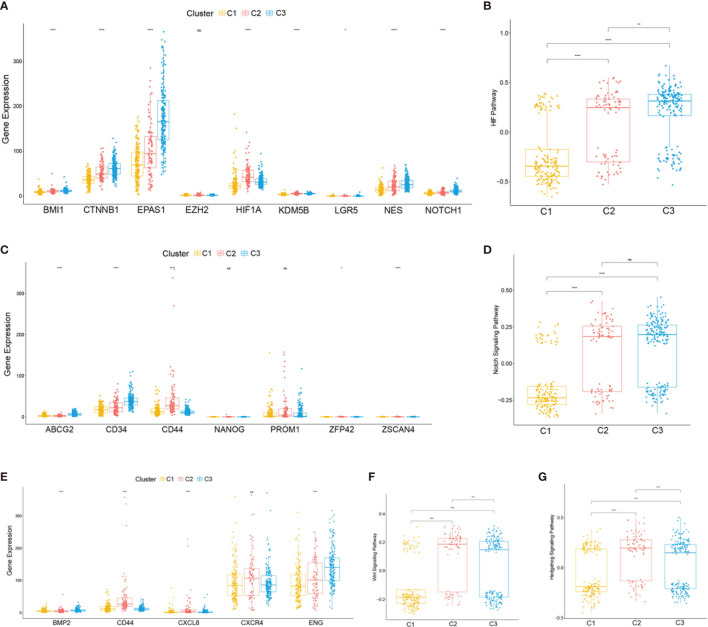
Expressions of clear cell renal cell carcinoma (ccRCC) stem cell-related genes and pathways. **(A, C, E)** Expression levels of cancer stem cell marker genes **(A)**, normal stem cell marker genes **(C)**, and ccRCC cancer stem cell marker genes **(E)**. **(B, D, F, G)** Box plot of the status of ccRCC cancer stem cell-related pathways. The *p*-values are labeled *above each box plot* with *asterisks*. *ns*, no significance. *p < 0.05, **p < 0.01, ***p < 0.001, ****p < 0.0001.

To further characterize the subclasses, the expressions of the RCSC surface markers (CD44, CXCRL8, CXCR4, and ENG) and the stem cell-related pathways such as hypoxia-inducible factor (HIF), Notch, Wnt, and Hedgehog were investigated. Subclass C2(SC_A) had the highest expression of RCSC marker genes (**Figure 4E**). Moreover, subclasses C2(SC_A) and C3(SC_D) had higher normal stem cell and CSC gene markers (**Figures 4A, C**). Generally, compared to that in subclasses C2(SC_A) and C3(SC_D), HIF, Notch, Wnt, and Hedgehog were less activated in subclass C1(SC_E) (**Figures 4B, D, F, G**). Besides, the immune and stromal scores of the subclasses were calculated using the ESTIMATE (estimation of stromal and immune cells in malignant tumours using expression data) algorithm. As shown in **Figures 3F, G**, subclass C2(SC_A) had the highest stromal and immune cell infiltration scores.

### Correlation of the ccRCC Subclasses With Immune Infiltration

**Figure 5:**
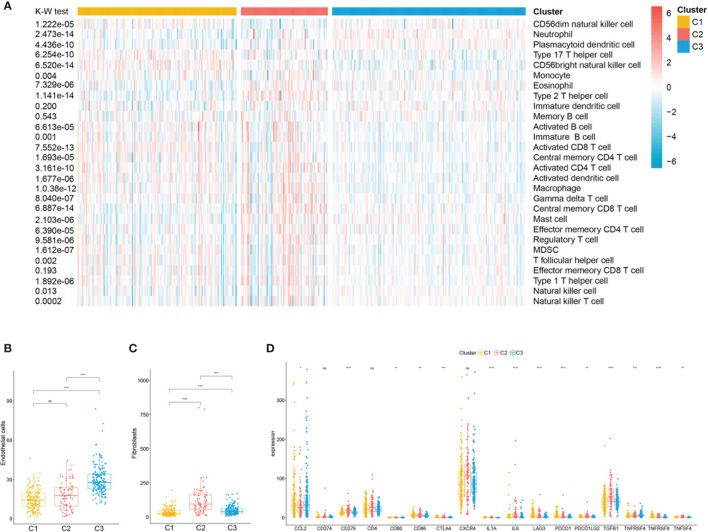
Immune characteristics of the three subclasses in The Cancer Genome Atlas (TCGA) training set. **(A)** Heatmap describing the abundance of immune cell populations in C1, C2, and C3. Box plot of the abundance of endothelial **(B)** and fibroblasts **(C)** distinguished by different subclasses. **(D)** Expression levels (in fragments per kilobase of transcript per million mapped reads, FPKM) of 17 immune checkpoint genes in the three clear cell renal cell carcinoma (ccRCC) subclasses. *ns*, no significance. *p < 0.05, **p < 0.01, ***p < 0.001, ****p < 0.0001.

Considering the significant differences in the immune and stromal scores displayed among the subclasses, immune and stromal cell infiltration was explored to depict their microenvironment landscape based on TCGA training cohort. Using the single-sample gene set enrichment analysis (ssGSEA) algorithm, the abundance rates of the 28 immune-related cell types were quantified and presented in a heatmap (**Figure 5A**). Subclass C2(SC_A) showed a significantly different immune cell infiltration compared with the other two subclasses. Stromal cells, especially fibroblasts, which have an important role in renal cancer progression, showed different infiltration status in the three subtypes (**Figures 5B, C** **)**. Moreover, we explored the association between subclasses and the expressions of potentially targetable immune checkpoint genes, which have been used for drug inhibitors in clinical trials or approved for specific cancer treatment (**Figure 5D**). Subclass C2(SC_A) had the highest expressions of most of these genes.

### Correlation of the ccRCC Subclasses With Mutations and Copy Number Variations

**Figure 6:**
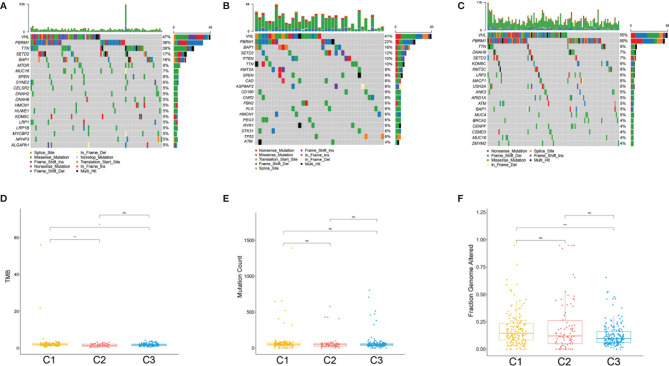
Association between the clear cell renal cell carcinoma (ccRCC) subclasses and mutations. **(A–C)** Oncoprint of the mutation status of the top 20 genes in subclasses C1(SC_E), C2(SC_A), C3(SC_D). **(D–F)** The tumor mutation burden **(D)**, mutation count **(E)**, and fraction genome altered **(F)** in the three subclasses. *ns*, no significance. *p < 0.05, **p < 0.01, ***p < 0.001.

The tumor genomic landscape has been demonstrated to be associated with antitumor immunity. To investigate whether differences exist in the somatic mutation frequencies across the ccRCC subclasses and to observe the different patterns of mutations among the ccRCC clusters, somatic mutation data from TCGA database were analyzed. **Figures 6A–C** show the landscape of the top 20 mutated genes in the three subtypes. There was no significant difference in the mutation count of the three subclasses (**Figure 6E**). However, the tumor mutation burden (TMB) (**Figure 6D**) and the fraction genome altered (**Figure 6F**) were significantly higher in subclass C1(SC_E) compared to those in subclasses C2(SC_A) and C3(SC_D).

### Transcriptome Features of the ccRCC Subclasses

**Figure 7:**
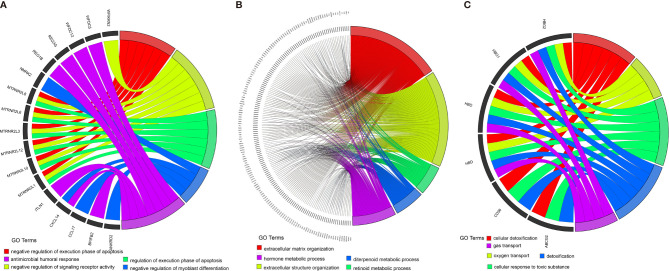
Enrichment analysis of the differentially expressed genes in three different subclasses: C1 **(A)**, C2 **(B)**, and C3 **(C)**.

To better characterize the three ccRCC subclasses, gene differential analysis was conducted. Genes with an adjusted *p*-value less than 0.01 and an absolute log2 fold change larger than 1 were considered significantly differential. Only those genes which showed significant differences in two possible comparisons were considered subclass-specific genes. Finally, all of the 1,695 subclass-specific genes were identified, including 172 specific genes for C1(SC_E), 1,485 specific genes for C2(SC_A), and 38 specific genes for C3(SC_D). Subsequently, gene ontology (GO) enrichment analysis of the subclass-specific genes was performed using clusterProfiler in the R package. The significantly enriched biological processes are shown in **Figure 7**. Subclass C2(SC_A), which was enriched in extracellular matrix organization and extracellular structure organization, had significantly different enriched pathways compared with the other subclasses (**Figure 7B**).

### Prediction of the Therapeutic Response of the ccRCC Stem Cell Subtypes to Immune Checkpoint Inhibitors and Target Therapy

**Figure 8:**
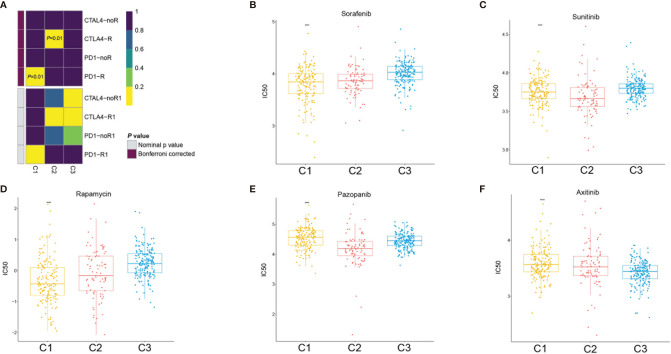
Immunotherapy and target therapy response prediction in the clear cell renal cell carcinoma (ccRCC) subclasses. **(A)** Response of the three ccRCC subclasses to PD-1 and CTLA-4 immunotherapy. **(B–F)** Sensitivity of the three ccRCC subclasses to sorafenib **(B)**, sunitinib **(C)**, rapamycin **(D)**, pazopanib **(E)**, and axitinib **(F)**. ****p < 0.0001.

Based on the above results, we further evaluated the response of the three subtypes to immunotherapy. In RCC, the blockade of PD-1 and CTLA-4 has become the new treatment approach in patients with intermediate- and high-risk metastatic tumors, whereas monotherapy with the PD-1 inhibitor nivolumab is the second-line or third-line treatment approach after failure of VEGF tyrosine kinase inhibitors (16). In 2019, the United States Food and Drug Administration (FDA) approved the combination use of PD-1 blockade and anti-angiogenic therapy for the treatment of patients with advanced RCC (17). To predict the sensitivity to immunotherapy of the different clusters, we performed subclass mapping to compare the expression profiles of the three stem cell subtypes with 47 melanoma patients who were treated with immunotherapy (18). The subclass mapping results indicated that the C2(SC_A) subtype might be more sensitive to anti-CTLA-4 treatments (**Figure 8A**).

Since VEGF receptor (VEGFR) target therapy is a more conventional therapy for patients with advanced ccRCC, we selected five conventional target therapy agents (sunitinib, sorafenib, axitinib, pazopanib, and rapamycin) and evaluated the responses of the three subtypes. We constructed a prediction model on the Genomics of Drug Sensitivity in Cancer (GDSC) cell line dataset using ridge regression and evaluated the prediction accuracy using 10-fold cross-validation. We estimated the half-maximal inhibitory concentration (IC_50_) of each sample in the training set based on the prediction models for the four target therapy agents. Regarding sunitinib and pazopanib, subclass C2(SC_A) was the most sensitive (**Figures 8C, E**), while for sorafenib, subclass C3(SC_D) had the worst sensitivity. Subclasses C1(SC_E) and C2(SC_A) showed similar sensitivity values (**Figure 8B**). Regarding axitinib, subclass C3(SC_D) was the most sensitive (**Figure 8F**), while for rapamycin, subclass C1(SC_E) was the most sensitive (**Figure 8D**).

### Construction of a Prognostic Model Based on Key Genes

**Figure 9:**
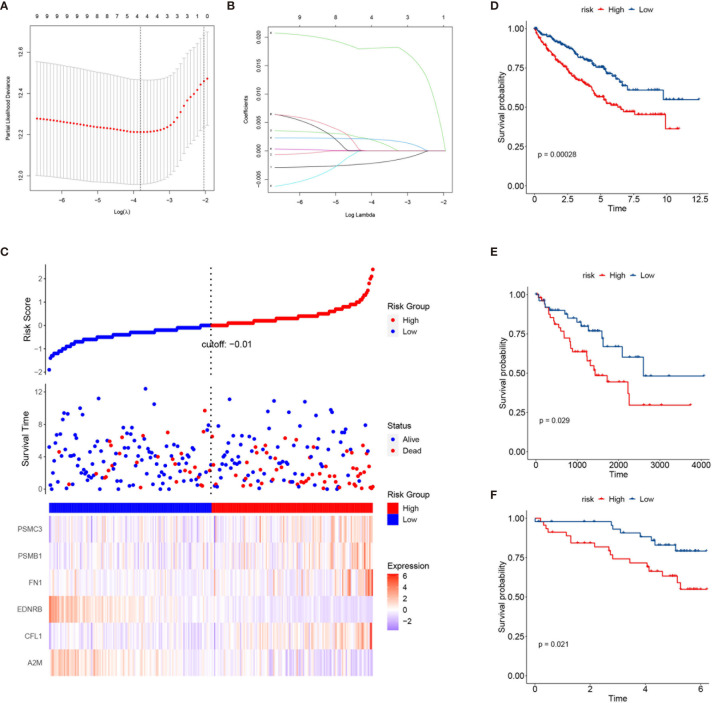
Construction of a risk prediction model based on the key genes in the three subclasses. **(A)** Tuning parameter (*λ*) screening in the least absolute shrinkage and selection operator (LASSO) regression model. **(B)** LASSO coefficient profiles of the common genes. **(C)** From *top to bottom* are the risk score distribution, survival overview, and heatmap analysis of six genes. **(D–F)** Kaplan–Meier plots of overall survival (OS) according to the risk scores in The Cancer Genome Atlas (TCGA) training set **(D)**, TCGA testing set **(E)**, and the International Cancer Genome Consortium (ICGC) cohort **(F)**.

To better characterize the prognosis of each patient, we constructed a risk model based on the key genes. The key genes were extracted from the stem cell-related genes using the NMF package in R. Twenty key genes were identified. Then, we applied least absolute shrinkage and selection operator (LASSO) Cox regression analysis to select the most useful predictive features and identified four genes (*A2M*, *CFL1*, *FN1*, and *PSME2*) with non-zero regression coefficients (**Figures 9A, B** **)**. Ultimately, a six-gene risk signature was built, and the risk score of each patient was calculated using the following formula:

Signature risk core = (−0.00205964493004206 × *A2M* expression) + (0.00157204565454328 × *CFL1* expression) + (0.00199772913455084 × *FN1* expression) + (0.0181057563160569 × *PSME2* expression). Increased expressions of *PSME2*, *FN1*, and *CFL1* correlated with higher risk scores and worse survival outcomes (**Figure 9C**). More importantly, the risk score could stratify patients into a high- and a low-risk group with significantly different survival outcomes (**Figure 9D**). Similar results were found in TCGA testing set and the ICGC dataset (**Figures 9E, F** **)**.

## Discussion

Although a variety of ccRCC classifications based on gene expression have been developed in recent years, a consensus in molecular subtype has not yet been achieved. To identify the ccRCC subgroups associated with CSC and patient prognosis, a ccRCC classification was developed in this study based on 392 genes retrieved from Molecular Signatures Database. Three subclasses of ccRCC with different prognosis were identified. Subsequently, the stem cell signature, immune infiltration, mutation landscape, and clinicopathological characteristics of the subclasses and their sensitivity to immunotherapy were investigated. The results showed that three subclasses were distinct, with significantly different stem cell and immune cell infiltration signatures. Drug sensitivity analysis demonstrated that subclass C2(SC_A) was sensitive toward CTLA-4 inhibitors and sunitinib. In addition, based on the marker genes of each cluster, we constructed a risk model to predict the prognosis of patients. This risk model could stratify patients with different prognosis, and it was validated in an external cohort.

The progression of cancer was accompanied by the gradual loss of differentiation ability and the gain of stem cell-like characteristics (12). Inhibiting the self-renewal capacity and the tumorigenicity of ccRCC significantly suppressed tumor growth and metastasis (19, 20). Given that a relapse in ccRCC has been attributed to the maintenance of ccRCC stemness cells, possessing stem cell properties that lead to therapy resistance (21), there is an urgent need for the development of prognostic biomarkers associated with stem cell properties. Malta et al. developed a novel transcriptome stemness index called mRNAsi (mRNA expression-based stemness index) to evaluate the stemness based on the one-class logistic regression machine learning algorithm (22). However, the mRNAsi was higher in normal renal tissue compared with that in renal tumor and showed no correlation with the survival outcomes of patients. This contradicts usual biological experiment results that demonstrate CSC properties to indicate worse prognosis. A novel stem cell-related prognostic evaluation model is needed.

Based on stem cell-related genes, three subtypes with different survival outcomes were identified. The results showed that subclass C1(SC_E) was distinct with the negative regulation of stem cell maintenance; however, subclasses C2(SC_A) and C3(SC_D) displayed distinct stem cell signatures and were characterized by positive regulation of stem cell maintenance. Moreover, subclass C2(SC_A) was also highly correlated with stem cell division and differentiation. The signature differences between subclasses C2(SC_A) and C3(SC_D) may be caused by the dormancy of CSCs (23, 24). Renal CSC surface markers, such as CD44, CXCR4, and CD105(ENG), were highly expressed in subclasses C2(SC_A) and C3(SC_D) (10, 11). BMP2 and CXCL8, which could promote the self-renewal of RCSCs, also had higher expressions in subclasses C2(SC_A) and C3(SC_D) (15, 25). The HIF, Notch, Wnt, and Hedgehog signaling pathways were reported to promote renal cancer progression by regulating the self-renewal and stemness maintenance of RCSCs (14, 26–28). Hence, we also investigated their status in the different subclasses. Compared to that in subclass C1(SC_E), these pathways were significantly activated in subclasses C2(SC_A) and C3(SC_D). These results demonstrated the validity of this classification. Moreover, these signaling pathways were also differently expressed in subclasses C2(SC_A) and C3(SC_D). These results indicated that these pathways were not only implicated in stemness maintenance but also associated with the proliferation and differentiation of RCSCs.

A recent study has demonstrated that stem cell properties are microenvironment defined during tumor progression (29, 30). Hence, we analyzed the stromal and immune infiltration levels in the three subtypes using R package ESTIMATE. The C2(SC_A) subtype had higher stromal and immune scores. Subsequent analysis further corroborated the C2(SC_A) subtype as possessing distinct stromal and immune features, including high infiltration of fibroblasts, T cells, and macrophages. Heterogeneous stromal cells in the tumor microenvironment can profoundly boost cancer progression (31). Carcinoma-associated fibroblasts (CAFs) form the chief components of the tumor microenvironment in multiple types of malignancies (32). By providing a supporting niche for CSCs, CAFs could facilitate tumor formation and induce chemoresistance (31). This may partly be explained by the concurrence of a high stem cell maintenance score and fibroblast infiltration. Macrophages abundantly exist in the immune milieu, where they share the microenvironment with CSCs. Macrophage-initiated WNT signaling could contribute to the maintenance of stemness, leading to the characteristics of chemoresistance and invasiveness in ovarian cancer (33). A similar phenomenon was found in lung cancer. A positive feedback interaction between macrophages and cancer cells could promote the stemness of cancer cells (34). Tumor-associated macrophages have been demonstrated to contribute to the maintenance of breast CSC populations through triggering the production of the inflammatory cytokines interleukin 1 (IL-1), IL-6, and IL-8, which, in turn, reinforce the CSC states (35). Interestingly, previous studies proved that IL-8 could boost the CSC-like properties of ccRCC (15). Besides, the activation of the Notch signaling pathway could promote the expansion of ccRCC-derived CSCs and induce chemotaxis simultaneously (27). Th17 cells are another type of immune cells that appear to support CSCs. Th17 cell-associated cytokines could transform dormant stem cells into an active state (36). This is consistent with our finding that subclass C2(SC_A) had a higher infiltration of Th17 helper cells. Tumor-specific antigens are usually generated by somatic mutation and can influence the response of patients to immunotherapy (37, 38). Hence, we comprehensively analyzed the mutation status of the three subclasses. Although there was no significant difference in the TMB among the three subtypes, C1(SC_E) had higher mutation counts and fraction genome altered than the other subclasses. These differences might influence their response to immunotherapy. *VHL* mutation is the most common mutation in ccRCC (39). However, evidence of the relationship between the mutation status of the *VHL* gene and ccRCC remains few and contradictory. Some studies suggested that *VHL* mutation could activate effector T cells and promote the secretion of cytokines (40). However, a recent study has proposed that wild-type *VHL* positively correlated with the expression of programmed death-ligand 1 (PD-L1) (41). In the present study, we found that subclass C2(SC_A) had a relatively lower *VHL* mutation frequency. *PBRM1* is another commonly mutated gene in ccRCC. A previous study indicated that CD8^+^ T-cell-infiltrated tumors had relatively fewer *PBRM1* mutations (42). Similarly, we found that the mutation frequency of *PBRM1* was significantly lower in subclass C2(SC_A), which had the highest immune cell infiltration. Moreover, the mutation landscape showed that subclass C1 had the highest mTOR mutation frequency. Correspondingly, patients in subclass C1 were most sensitive to the mTOR inhibitor rapamycin. This demonstrated the reliability of the present study. More studies are needed to investigate the relationship between somatic mutations and immune infiltration.

Considering the close interactions between CSCs and the immune system, developing therapeutic strategies that target immune checkpoints might pave the way to eradicating CSCs. At present, immunotherapy has obtained global attention in cancer management. The efficacy and safety of PD-1 immune checkpoint inhibitors and CTLA-4 inhibitors have been applied clinically and have shown promising outcomes (43, 44). Due to the interaction between PD-1 and PD-L1 in tumor-infiltrating lymphocytes and tumor cells, T-cell exhaustion, tumor-specific T-cell dysfunction, and immune evasion by tumor cells were triggered. Exhausted T cells could produce additional inhibitory molecules to promote the progression of cancer; however, this process could be reversed by a combined PD-1 and CTLA-4 blockade. Treating a mouse tumor model with PD-L1 and CTLA-4 inhibitors could promote the elimination of CSCs (45).

Redirecting immune suppression by targeting checkpoints has brought about clinical response in some RCC patients, and a combination treatment involving checkpoint blockade is now the standard of therapy in advanced RCC patients. However, a substantial subset of patients is not sensitive to checkpoint blockade. The identification of a reliable evaluation system to predict the response to checkpoint blockade is essential to improve the clinical efficacy of these therapies (46). Moreover, the immune checkpoint gene *CXCR4*, which is also a marker of RCSC, was highly expressed in subclass C2(SC_A). Targeting the tumor microenvironment may provide a promising therapeutic avenue as the eliminated CSCs could be replenished by non-CSCs for the existence of a survival niche (47). The highest expressions of immune checkpoint genes indicated the sensitivity of subclass C2(SC_A) to immunotherapy. The results demonstrated that anti-CTLA-4 therapy and sunitinib are promising for patients in subclass C2(SC_A). The results of our study provide a novel insight into the combination of anti-CTLA-4 therapy and sunitinib, which requires further validation in future research.

Besides, to better characterize the prognosis of each patient, we constructed a risk model based on the key genes (*A2M*, *CFL1*, *FN1*, and *PSME2*). The risk scores could classify patients into the high- and low-risk groups with significantly different survival outcomes. In addition, the effectiveness of this risk model was validated in TCGA testing set and the ICGC dataset.

In summary, in this study, we explored the stem cell-related process landscape of ccRCC and identified three subclasses with different stem cell activities. We systematically analyzed the differences of these subclasses in the tumor microenvironment, immune cell infiltration, immunotherapy/target therapy response, and the corresponding pathways and constructed a risk model. The results of this study provide a basis and reference for the treatment and prognostic prediction of ccRCC.

With the development of target therapy and immunotherapy, various drugs have been used for the clinical treatment of advanced ccRCC. The VEGFR inhibitor, anti-checkpoint therapy, and the combination of both showed promising efficacy (48, 49). However, few references were established for the selection of a proper treatment plan. The novel developed stem cell subtypes are critical for selecting suitable therapies for ccRCC patients. Patients in the stem cell activated subtype (SC-A) could benefit more from anti-CTLA-4 and sunitinib treatment. For those in the stem cell excluded subtype, an anti-PD-1 therapy might be more suitable. However, there are limitations in the present study. This study was conducted based on an existing public dataset. These findings need to be validated in larger ccRCC patient cohorts with immunotherapy and target therapy experience.

## Materials and Methods

### Patients and Samples

The RNA sequencing data (raw counts) of 530 and 91 ccRCC patient samples with corresponding clinical information were download from The Cancer Genome Atlas (TCGA; http://cancergenome.nih.gov/) and the International Cancer Genome Consortium (ICGC; www.icgc.org), respectively. Patients with 0 day overall survival (OS) were removed; 526 TCGA samples were retained for further analysis. Subsequently, the dataset from TCGA was randomly divided into a training set and a testing set. The gene expression data of 91 ccRCC samples from the ICGC were used for external validation. In total, 662 ccRCC patients were enrolled in the present study. The gene somatic mutation data (MAF files) of ccRCC were retrieved from TCGA.

### Identification of ccRCC Subclasses

Human stem cell-related biological processes were downloaded from the Molecular Signatures Database (https://www.gsea-msigdb.org/gsea/msigdb/index.jsp). A total of 392 genes from 19 stem cell-related biological processes were obtained (**Supplementary Table S1**). These genes were FPKM (fragments per kilobase of transcript per million mapped reads) normalized and used for subsequent non-negative matrix factorization (NMF) analysis using the R NMF package in the training set. NMF is an unsupervised learning technique that has been used to extract meaningful information from high-dimensional data (50). In detail, the best *k*-value was defined according to the cophenetic correlation coefficients, dispersion, and silhouette. The iteration was set as *n*_run_ = 100. This method was also applied to the testing and external validation sets using the same candidate genes. The first value of *k* for which the cophenetic coefficient starts decreasing was chosen as the optimal number for clustering. A *t*-distributed stochastic neighbor embedding (t-SNE)-based approach was then used to validate the sample clustering using the mRNA expression data of the above stem cell-related genes.

### Gene Set Variation Analysis

Gene set variation analysis (GSVA) is a non-parametric and unsupervised gene set enrichment method that can estimate the scores of certain pathways or signatures based on transcriptomic data. Using the GSVA R package, each sample received 19 scores corresponding to 19 stem cell-related signatures. Subsequently, differences in the signatures of the different clusters were calculated using the *t*-test in R.

### Estimation of Immune Infiltration

The microenvironment cell populations counter (MCP-counter), a method based on transcriptomic data, was used to assess the absolute abundance of two stromal cell populations (endothelial cells and fibroblasts) (51). Furthermore, another approach applied for the qualification of the immune infiltration of 28 immune cells used in this research was single-sample gene set enrichment analysis (ssGSEA), which calculated an enrichment score (ES) representing the variations of the pathway activities within a single sample (51). In addition, immune scores and stromal scores were calculated using the ESTIMATE algorithm, which can reflect the infiltration level of stromal and immune cells.

### Enrichment Analysis

Kyoto Encyclopedia of Genes and Genomes (KEGG) pathway enrichment and Gene Ontology (GO) analysis were performed with the R package “clusterProfiler.”

### Target Therapy and Immunotherapy Sensitivity Prediction

The sensitivity of patients to target therapy drugs was evaluated based on the GDSC database (https://www.cancerrxgene.org/) (52). IC_50_ values were estimated using the R package pRRophetic (53). In detail, the IC_50_ was calculated by ridge regression and the prediction accuracy was evaluated using 10-fold cross-validation based on the GDSC training set. The response to anti-PD-1 and anti-CTLA-4 therapy was predicted by comparing the expression profiles of three subtypes with 47 melanoma patients who respond to the immunotherapy using subclass mapping (https://www.genepattern.org/).

### Construction of a Prognostic Model

Univariate Cox regression was used to screen the mRNAs affecting the OS of patients (*p* < 0.05). Thereafter, survival-related genes were screened with the LASSO multivariate Cox regression algorithm using the R package “glmnet” (version 3.0). Finally, the signature genes and coefficients in the risk score signature were constructed based on the most proper penalty parameter *λ*. The risk score formula used was:


$$\text{Risk score}=\Sigma_{i=1}^{n}\;\text{Coe}{\text{f}}_{i}*\text{Ex}{\text{p}}_{i}$$

where Coef*_i_* is the coefficient and Exp*_i_* is the normalized expression of each gene in the signature. The risk score system was constructed using the training set and evaluated in the testing set. Patients were stratified into a high-risk group and a low-risk group based on the median risk score.

### Statistical Analysis

All statistical analysis was performed using R programming (https://www.r-project.org/). Unpaired Student’s *t*-test was used to compare two groups with normally distributed variables. For the comparison of three groups, one-way analysis of variance and the Kruskal–Wallis test were used as parametric and non-parametric methods, respectively. Contingency table variables were analyzed with the chi-square test or Fisher’s exact tests. Survival analysis was carried out using the Kaplan–Meier method and compared using the log-rank test. A *p*-value less than 0.05 was considered as statistically significant.

## Data Availability Statement

The original contributions presented in the study are included in the article/**Supplementary Material**. Further inquiries can be directed to the corresponding author.

## Author Contributions

HW designed the study and drafted the manuscript. HW and HX collected, analyzed, and interpreted the data. QC and CL participated in revising the manuscript. All authors contributed to the article and approved the submitted version.

## Funding

This work was supported by Research Fund of Anhui Institute of Translational Medicine (grant number: ZHYX2020A003).

## Conflict of Interest

The authors declare that the research was conducted in the absence of any commercial or financial relationships that could be construed as a potential conflict of interest.

## Publisher’s Note

All claims expressed in this article are solely those of the authors and do not necessarily represent those of their affiliated organizations, or those of the publisher, the editors and the reviewers. Any product that may be evaluated in this article, or claim that may be made by its manufacturer, is not guaranteed or endorsed by the publisher.

## Abbreviations

ccRCC, clear cell renal cell carcinoma; CSC, cancer stem cell; SC-A, stem cell activated; SC-D, stem cell dormant; SC-E: stem cell excluded; OS: overall survival; TMB: tumor mutation burden.
